# Knowledge Graphs for Indication Expansion: An Explainable Target-Disease Prediction Method

**DOI:** 10.3389/fgene.2022.814093

**Published:** 2022-03-14

**Authors:** Ozge Gurbuz, Gregorio Alanis-Lobato, Sergio Picart-Armada, Miao Sun, Christian Haslinger, Nathan Lawless, Francesc Fernandez-Albert

**Affiliations:** ^1^ Discovery Research Coordination Germany, Boehringer Ingelheim Pharma GmbH & Co. KG, Biberach an der Riss, Germany; ^2^ Global Computational Biology and Data Sciences, Boehringer Ingelheim Pharma GmbH & Co. KG, Biberach an der Riss, Germany

**Keywords:** knowledge graphs, ontologies, drug discovery, target repurposing, target repositioning

## Abstract

Indication expansion aims to find new indications for existing targets in order to accelerate the process of launching a new drug for a disease on the market. The rapid increase in data types and data sources for computational drug discovery has fostered the use of semantic knowledge graphs (KGs) for indication expansion through target centric approaches, or in other words, target repositioning. Previously, we developed a novel method to construct a KG for indication expansion studies, with the aim of finding and justifying alternative indications for a target gene of interest. In contrast to other KGs, ours combines human-curated full-text literature and gene expression data from biomedical databases to encode relationships between genes, diseases, and tissues. Here, we assessed the suitability of our KG for explainable target-disease link prediction using a glass-box approach. To evaluate the predictive power of our KG, we applied shortest path with tissue information- and embedding-based prediction methods to a graph constructed with information published before or during 2010. We also obtained random baselines by applying the shortest path predictive methods to KGs with randomly shuffled node labels. Then, we evaluated the accuracy of the top predictions using gene-disease links reported after 2010. In addition, we investigated the contribution of the KG’s tissue expression entity to the prediction performance. Our experiments showed that shortest path-based methods significantly outperform the random baselines and embedding-based methods outperform the shortest path predictions. Importantly, removing the tissue expression entity from the KG severely impacts the quality of the predictions, especially those produced by the embedding approaches. Finally, since the interpretability of the predictions is crucial in indication expansion, we highlight the advantages of our glass-box model through the examination of example candidate target-disease predictions.

## Introduction

Indication expansion (IE) is an emerging subject in drug discovery that aims to find alternative therapeutic applications, or diseases (indications) for an existing drug target (Parisi et al., 2020). Considering the high cost and slow process of bringing a new drug into the market, *in silico* approaches for drug discovery and repurposing (Dudley et al., 2011; Picart-Armada et al., 2019; Sosa et al., 2020) became a popular subject in the bioinformatics community due to the increasing availability of both structured and unstructured data modalities. In fact, with the improvement in text mining technologies, literature mining has become an established and popular tool for indication expansion in drug discovery (Andronis et al., 2011; Lekka et al., 2012; Smalheiser, 2012; Sebastian et al., 2017; Sang et al., 2018; Sosa et al., 2020). One can search for all potential disease relations for a given drug in the literature via text mining techniques and expand the analysis to all targets of the drug to establish a more comprehensive search (Andronis et al., 2011; Lekka et al., 2012; Smalheiser, 2012). The outcome of this method is the direct disease-gene links (based on search criteria). On the other hand, analysis of biological data sources (such as molecular data, experimental data, gene expression data, etc.) are common approaches to search for novel target-disease links (Brown and Patel, 2017; Härtner et al., 2018; Picart-Armada et al., 2019).

A natural extension of these studies would be the integration of several data sources for a more comprehensive analysis. However, the heterogeneity of data formats and sources raises questions during their integration (Holzinger, 2018; Katsila and Matsoukas, 2018). The best way to undertake this data integration challenge, together with data contextualization, is the application of semantic web technologies: ontologies and knowledge graphs (Qu et al., 2009; Chen and Xie, 2010; Williams et al., 2012; Lin et al., 2017; Kanza and Frey, 2019; Zhu et al., 2020). The main ideas of ontologies and knowledge graphs (KGs) are that each resource has a unique identifier, and once each resource is defined with the identifier, regardless of where they are extracted from, they will be merged and the integration process will be effortless. Secondly, integrating the data sources brings up the topic of data governance, as data needs to be findable, accessible, interoperable, and reusable or, in other words, in alignment with the FAIR data principles (Wilkinson et al., 2016). For this, ontologies can also be very helpful (Williams et al., 2012) because all the data mapped using the same ontology will be already linked which makes it very easy to search, query, and reuse. Lastly, predictions from comprehensive and integrated data sets are often difficult to interpret (Holzinger, 2018; Lecue, 2020). This is a major challenge in the biological domain, which can be tackled by providing ontological perspective into the prediction process to incorporate human recognition and interpretation, thus making the methodology a “glass box” model (Holzinger et al., 2017). Due to the importance of semantic web technologies in addressing the above-mentioned challenges, many researchers have added a semantic layer and included KGs in their computational methods for drug discovery studies (Kanza and Frey, 2019).

In our previous work, we divided the studies that use KGs for drug discovery into two categories (Gurbuz et al., 2020): KGs built from biological data sources (Qu et al., 2009; Fu et al., 2016; Han et al., 2018; Celebi et al., 2019; Zhu et al., 2020) and KGs built from the literature (Sang et al., 2018, 2019; Sosa et al., 2020). Then, we distinguished between studies performing drug-disease predictions (Qu et al., 2009; Fu et al., 2016; Han et al., 2018; Sang et al., 2018, 2019; Sosa et al., 2020; Zhu et al., 2020) and those predicting drug-drug interactions (Herrero-Zazo et al., 2015; Celebi et al., 2019). The outcome of this review of the state-of-the-art was that the studies using structured biological data sources (BioGrid, StringDB, Human Protein Atlas, etc.) for building the KG applied several network analysis methods to predict either drug-disease relations or gene-disease associations. Even though the value of available biological sources cannot be denied, the outcome of such predictions based on statistical confidence scores may not be sufficiently persuasive to kick-off a full drug-development program (Holzinger, 2018). Literature support would be more convincing for further investigation. Therefore, the second group of studies constructed the KG from literature sources but did not implement KGs that combine both structured biological data sources and literature sources for a more comprehensive indication expansion or target repositioning approach. Additionally, all these studies did not truly benefit from semantic web technologies. Instead, they directly applied network analysis algorithms.

As a result, in the past we conducted an exploratory case study aimed at constructing a comprehensive KG to facilitate indication expansion (Gurbuz et al., 2020). We presented the methodology, defined the reasoning and inferencing on the KG, and successfully applied it to two randomly selected cases to predict the link between the target and disease. We ranked the paths connecting the target and disease based on the number of publications associated with its constituent edges. In addition, a path was considered more relevant when all the proteins in the path showed expression in the same tissue, either at the RNA or protein level. One limitation of the previous study was that we conducted the exploratory cases at a small scale with a target and a given candidate indication to find the mechanism of action. By contrast, in the current study we extend the identification of novel target-disease links to all available pairs, evaluate the performance of the inferred edges based on random baselines, and study the value of including RNA- and protein-level expression information in our predictions. Moreover, we show how the KG can be exploited to interpret candidate gene-disease associations through the examination of two examples.

## Related Work

In this section, we review the approaches that have resorted to the use of KGs for drug discovery regardless of whether the purpose was drug-disease, gene-disease, or drug-drug interaction prediction. Table 1 shows a comprehensive overview of the reviewed methods.

**TABLE 1:** Overview of knowledge graph usage in drug discovery.

Study	Purpose	Method	Data source
Celebi et al. (2019)	Drug-Drug Interaction: evaluating the different embedding methods in various Cross Validation schemes	Embedding: RDF2Vec, CBOW, Skip Gram, TransE, TransD ML Model: Logistic Regression, Naive Bayes, Random Forest	Bio2RDF
Fu et al. (2016)	Drug-target interactions	Metapath + Random forest, SVM	Biological and chemical datasets
Han et al. (2018)	Drug target genes for Alzheimer’s Disease	Inference + enrichment analysis	TTD, DrugBank, PharmGKB, AlzGene
Zhu et al. (2020)	Drug centric KG	Positive and Unlabeled Learning * SVM, Decision Tree and Random Forest	PharmGKB, TTD, KEGG DRUG, DrugBank, SIDER and DID
Sang et al. (2018)	Potential drugs for diseases	Logistic regression	Pubmed Abstracts
Sang et al. (2019)	Potential drugs for diseases	TransE embedding + LSTM	Pubmed Abstracts
Sosa et al. (2020)	FDA approved drugs for rare diseases	Network proximity	Pubmed Abstracts
Paliwal et al. (2020).	Predicting clinical failure	Tensor factorization + gene prioritization	20% is from biomedical literature and biological data sources
Nunes et al. (2020)	Predicting Gene-Disease links	Embeddings + Random Forest	Gene-Disease links from Disgenet
Geleta et al. (2021)	Knowledge Graph construction to support drug discovery like predicting Gene-Disease links and	Embeddings (RESCAL) + XGBoot	Gene and Disease nodes and edges from public databases
KG for IE	Target repurposing	Tissue based semantic inferencing + Embeddings & Random Forest	Human curated full text literature + biological database

Celebi et al. used KGs for drug-drug interaction identification and used a publicly available dataset called Bio2RDF to extract drug features (Celebi et al., 2019). After feature extraction via RDF2Vec, TransE, and TransD embeddings, they applied Logistic Regression, Naïve Bayes, and Random Forest models and evaluated which combination of embedding and machine learning models was better at predicting a reference set of drug-drug interactions. The best performance they achieved was using RDF2Vec together with a Random Forest model.

There are several studies which use KGs to predict drug-disease relations. Fu et al. (Fu et al., 2016) built a network from various biological and chemical data sources to predict drug-target relations using Random Forest and Support Vector machine algorithms. However, they only benefited from semantic web technologies at the stage of data integration and concentrated on drug-target relations. Han et al. (Han et al., 2018) integrates popular biological databases (DB) such as TTD, DrugBank, PharmGKB, and AlzGene to predict novel drug targets for Alzheimer’s disease. Their novel strategy was to combine ontology inference together with enrichment analysis. However, their main goal was limited to finding genes for one specific disease, Alzheimer’s in this case. Zhu et al. built a drug centric KG by integrating six drug data sources (PharmGKB, TTD, KEGG DRUG, DrugBank, SIDER, and DID) (Zhu et al., 2020). They implemented a machine learning approach on a path-based representation and an embedding-based representation, separately. To evaluate the effectiveness of the KG, the authors used positive samples and unlabeled samples (samples from diabetes mellitus only) and implemented positive and unlabeled learning (PU) with Decision Tree, Random forest, and SVM models. According to their performance evaluations, the best outcome came from SVM implemented on path-based representation (normalized path count). However, this study uses a drug centric KG to understand the drug-disease interaction.

On the other hand, there are two studies by Sang et al. (Sang et al., 2018; Sang et al., 2019) which used literature for building the KG including SemaTyp. This KG is built from PubMed abstracts by using a natural language processing (NLP) tool called SemaRep. In the first study (Sang et al., 2018), they applied logistic regression on the KG and outperformed the results obtained with a random walk method. Their aim was to predict drug-disease relations via drug-target-disease chains. Later, the authors published a continuation of their work called GrEDeL in which they used KG embedding methods for discovering drug-disease relations from literature (Sang et al., 2019). The authors again use SemaRep to extract associations from PubMed abstracts and build the KG. This time they claim that their previous work, which used logistic regression, couldn’t reflect the order of the entities in the associations and couldn’t show the detailed drug mechanism of action. Therefore, they first used the TransE embedding method and applied a Long Short-Term Memory (LSTM) based Recurrent Neural Network model to show that graph embeddings capture more information than logistic regression. However, they claimed that the limitation of both studies is that the effectiveness of the methods is dependent on the NLP tool. Likewise, Sosa et al. (Sosa et al., 2020) also constructed a KG from PubMed abstracts to repurpose FDA-approved drugs for rare diseases. They used graph embedding and network proximity for generating their hypothesis. The limitation of this study is that they missed important knowledge that is usually present in the full text but not in the abstract. Moreover, Nunes et al. (Nunes et al., 2020) implemented a KG using all curated gene-disease links extracted from DisGeNET^1^. They filtered out genes that did not have protein correspondence in Uniprot or annotations in the Gene ontology and genes and diseases that were not annotated in Human Phenotypes. Then, they created 3 different KGs based on this filtering and deployed several embedding strategies, noting that they achieved their best performance for predicting gene-disease links with OPA2Vec. However, they only included gene-disease relations in their KGs.

Furthermore, in another study by Paliwal et al. (Paliwal et al., 2020), the authors built a heterogeneous KG in which 20% of the data comes from biomedical literature databases and the rest from biological data sources. These sources consist of entities such as genes, proteins, diseases, gene ontology processes, pathways, and compounds (Paliwal et al., 2020). Although the aim of the study was to evaluate translatability of *in silico* predictions of clinical trial failure, they were able to predict therapeutic genes for diseases using gene prioritization algorithms. Note that, contrary to what we do in this work, Paliwal and colleagues searched for therapeutic genes related to a group of selected diseases. A similar study from Geleta et al. (Geleta et al., 2021) also presents a comprehensive knowledge graph built from internal data, external public databases such as ChEMBL and Ensembl, and information extracted from PubMed full-text using Natural Language Processing Techniques named Biological Insights Knowledge Graph (BIKG) to be used for knowledge discovery with machine learning. They use RESCAL for knowledge graph embeddings and XGBoost as machine learning method. They report their average F1 score as 88% for gene-disease link prediction where they reduce the size of the KG to Gene and Disease nodes. However, their focus lies on the creation of the KG, whereas our paper addresses the practical utility of KGs in the context of indication expansion in drug development. Furthermore, they depend on natural language parsers and the full details about the method for gene-disease prediction are unavailable, hindering their reproducibility and application to drug discovery. Likewise, Ochoa et al. (Ochoa et al., 2020) also present a comprehensive knowledge graph with characterization of targets, diseases, phenotypes, and drugs to support target identification and prioritization. This is part of an update within the Open Targets platform. While full text literature is a data stream within Open Targets, its use for drug discovery in indication expansion is not explored.

After analyzing these studies, we concluded that KGs are becoming mainstream for supporting drug discovery initiatives, but they have not benefited from semantic information and instead have relied directly on the application of network analysis. In consequence, we evaluated both tissue-based semantic inferencing and various embedding strategies. Additionally, most literature-based KGs were constructed with abstracts. However, the authors behind these studies have acknowledged that this is a limitation and that extracting information from full texts would increase the predictive power of KGs in general. Therefore, we set out to address these pitfalls and used full-text literature for building a KG for IE. Predictions based on this graph can be accompanied by the literature references supporting them, as well as the mechanisms of action. Furthermore, our KG takes tissue specificity information into consideration when inferencing and predicting target-disease links.

## Methodology

### Knowledge Graph Development for Indication Expansion

This section summarizes the methodology that improves upon the KG developed in our previous work to facilitate indication expansion studies. More details can be found in Gurbuz et al. (2020).

**FIGURE 1:**
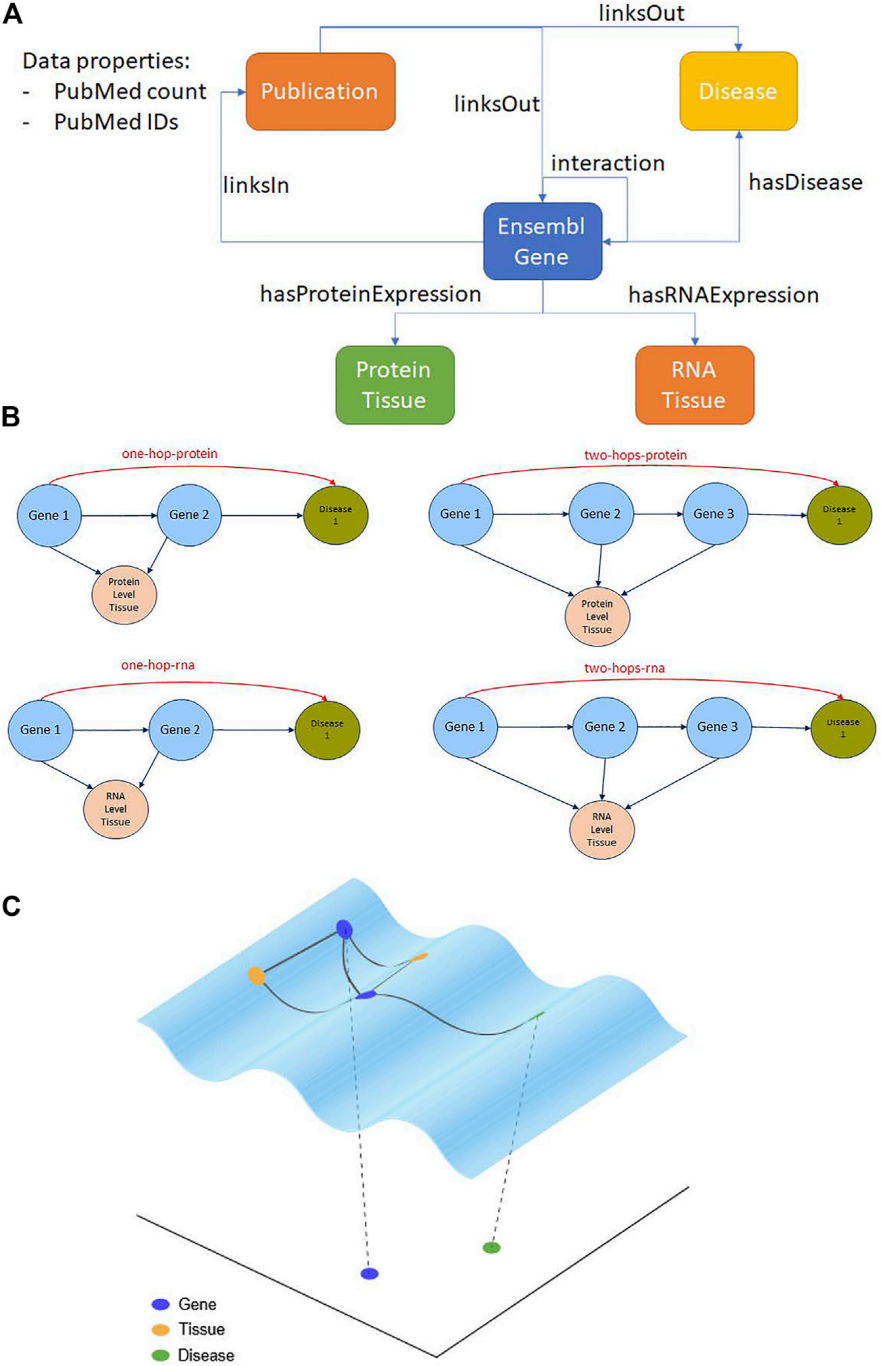
Knowledge graph schema and gene-disease prediction strategies. **(A)** Upper layer ontology with the entities and relations defining the structure and content of our knowledge graph. **(B)** Hop-based prediction strategies to find novel gene-disease associations via intermediary genes expressed in the same tissue at the RNA or protein levels. **(C)** Embedding-based prediction strategy to find novel gene-disease associations via distances/similarities in a latent space.

We start with the upper layer ontology, which defines the data and semantic layer of the KG. In the current study, we have improved the KG and included the following entities: Protein/Gene, RNA Tissue, Protein Tissue, Publication, and Disease. Figure 1A shows the updated upper layer ontology. We have used Python’s RDFLib^2^ for creating the ontologies and RDF graph. Since it was not possible to create edge properties with the RDF syntax and reification brings about efficiency problems, the new RDF* syntax can be used for creating a weight property on the edges (relations) with RDF4J ^3^. Alternatively, a third entity can be created to store the references of these genes’ connection information. In this study, we chose to create a third entity, named Publication, between gene and disease. This entity holds the information for PubMed IDs and the number of PubMed articles between the given gene and the disease as data properties.

After building the upper layer ontology, we populated it with Metabase^4^, a commercial source for human curated full-text literature information. We only selected the *high confident* relations between gene-gene and gene-disease interactions provided by Metabase. We extracted the tissue-level gene expression from the Human Protein Atlas^5^ (Uhlen et al., 2010). The pipeline for building and analyzing the KG is shown in Supplementary Figure S1. For data extraction and analysis, we used the R programming language and for ontology population and KG implementation we used Python’s RDFlib. Both data extraction and ontology population processes were automated with R and Python scripts (see script KGbuild_toy.py, which can be used as a template for KG construction). Therefore, building the KG took less than 1 day. We used Ensembl IDs for gene/proteins and Mesh IDs for diseases as Unique Resource Identifiers (URI).

### Characterization of the Knowledge Graph

We described the following topological features of the KG: in- and out-degree (i.e., number of directed links going in and out of a node, respectively), total degree (sum of in- and out-degree), edge density (ratio of the number of edges and the number of possible edges), value of the coefficient of the power-law distribution fitted to the degree distribution, and PageRank centrality (Page et al., 1999).

On the other hand, we explored the changes of the KG over the years, focusing on the largest (weakly) connected component consisting of only gene and disease nodes. To build the KG of a given year, we only kept the gene-gene and gene-disease edges whose first mention in literature was no later than that year. Then, we represented the evolution of the number of nodes, edges, the edge density, and the power law coefficient.

### Tissue-Based Gene-Disease Link Prediction From the Knowledge Graph

Since there may be indirect links (Lekka et al., 2012) between a gene and a disease *via* secondary signaling cascades (modelled as protein-protein interaction networks and pathways in our KG), we defined hop-based inferencing rules with RNA- and protein-level expression in tissues as key components (Gurbuz et al., 2020). For instance, say a protein/gene instance X interacts with another protein/gene instance Y and these two entities are expressed in the same tissues. Then, it is assumed that the disease D that Y is related to is one-hop-related to the instance X. Similarly, if protein/gene X interacts with Y, Y interacts with another protein/gene Z, all these entities are expressed in the same tissues and Z is associated with the disease D, we say that D is two-hop-related to the instance X (see Figure 1B). These candidate gene-disease links can be ranked according to the total number of publications in the X-Y-D or X-Y-Z-D path (i.e., the sum of the edge weights). A sample mock-up diagram can be found in Supplementary Figure S2.

For inferencing, we used five different strategies: one-hop links filtered by protein expression in the tissues, one-hop links filtered by RNA expression in the tissues, two-hop links filtered by protein expression in the tissues, two-hop links filtered by RNA expression in the tissues, and the union of all these types of predictions (see Figure 1B). We also evaluated the performance of the one- and two-hop strategies without the tissue filters.

### Random Baselines

We created 100 random KGs to evaluate the performance of the hop-based predictions. To this end, we shuffled the identity of the protein/gene entities, which maintained the structure of the KG unchanged but affected the biology encoded by the gene-gene, gene-disease, and gene-tissue components of the graph.

### *In Silico* Validation of Tissue-Based Gene-Disease Predictions

For each gene-gene and gene-disease link, we have the information of when the association was first reported (published) and what is the last record (publication) of such association. Accordingly, we used a prospective time-split validation scheme, where interactions and indications published before or in 2010 were eligible for the training data, whereas indications reported after 2010 were used to construct a gold standard or test set (see Table 2). It is important to note that the gold standard was constructed by making sure that only genes and diseases which also exists in KG_Before2010 were included, as these are the only cases that can be predicted. We further refined the gold standard by removing gene-disease pairs separated by more than two hops in the original KG. This led to fairer performance metrics because we considered a maximum of two hops of separation between genes and diseases in our predictions. Therefore, the final test set comprised 5,176 reference gene-disease associations.

**TABLE 2:** Validation scheme based on the date when the interaction was first reported.

Node1	Node2	Interaction	First referenced	Graph	Type
Gi	Dk	hasDisease	≤2010	KG_Before2010	Train data
Gi	Gj	activates	≤2010	KG_Before2010	Train data
Gj	Dk	hasDisease	>2010	KG_After2010	Test data

### Knowledge Graph Embeddings

We employed the Nunes et al. (2020) implementation of the most commonly used embedding methods for KGs, which are RDF2Vec^6^, DistMult^7^, TransE^8^, TransH^9^, and TransD^10^ to embed KG_Before2010 into a low-dimensional space (see Figure 1C). We used a 200-dimensional space as recommended in Nunes et al. (2020). Therefore, we obtained 200-dimensional representations of all the gene and disease entities, which we used to calculate Euclidean distances and cosine similarities between gene-gene and gene-disease pairs. These distances/similarities were used to build a Random Forest model that we applied to the prediction of gene-disease links. We selected this machine learning approach based on the work of Celebi et al. (2019) and Nunes et al. (2020) who found that Random Forests outperformed other techniques in their studies for predicting gene-disease links from ontologies.

To train the Random Forest and evaluate its performance, we labeled all the gene-disease pairs separated by at most 2-hops and which did not take place in the train and test data (Table 2) as negative cases. This allowed to construct a training set (98,426 positive and 98,426 negative cases) and a test set (5,176 positive and 5,176 negative cases).

### Performance Metrics

We evaluated the overall prediction accuracy of the inference strategies described above using the following definitions:

• True positive: Gene-disease link inferred from KG_Before2010 and that is listed in the KG_After2010 gold standard.
• False positive: Gene-disease link inferred from KG_Before2010 but that is not listed in the KG_After2010 gold standard.
• False negative: Gene-disease link not inferred from KG_Before2010 but that is listed in the KG_After2010 gold standard.

In addition, we constructed a table with all the possible gene-disease links that can be formed with the KG_Before2010 data (18,045 unique genes and 330 unique diseases for a total of 5,954,850 possible gene-disease associations). This list was further reduced to gene-disease pairs separated by at most two hops in the KG_Before2010 for a total of 458,640. Then, we determined which of those combinations were corroborated in the gold standard (positive cases) and scanned the list decreasingly based on the scores assigned to each pair by the hop-based prediction strategies (see Figure 1B). Gene-disease links not predicted by the hop-based methods were given a score of 0. This allowed us to construct Receiving Operating Characteristic (ROC) and Precision-Recall curves (Cannistraci et al., 2013) using the following definitions:

• True positive: Gene-disease link above current weight threshold that was reported after 2010.
• False positive: Gene-disease link above current weight threshold that was not reported after 2010.
• False negative: Gene-disease link below current weight threshold that was reported after 2010.
• True negative: Gene-disease link below current weight threshold that was not reported after 2010.

### Data and Code Availability

Gene-gene links and gene-disease links were extracted from the commercial database Metabase^11^, which prevents us from sharing these data. However, the code we used to define and populate our KG is available in the github link: https://github.com/bi-compbio/kg_for_ie and can be used with publicly available databases like StringDB^12^ for gene-gene links and DisGeNET^13^ for gene-disease links. Gene-tissue links for the resulting KG can be retrieved using the Human Protein Atlas R package^14^ (Tran et al., 2019).

## Results

### Characterization of the Graph

The KG was created from 18,790 unique nodes (464 diseases; 18,165 genes; 124 ProteinTissues; 37 RNATissues) and 669,900 edges (70,380 hasDisease; 263,106 hasProteinExpression; 234,294 hasRNAExpression; 102,120 Interaction). The graph was directed and contained no multi-edges. After imposing the publication date restriction, *KG_Before2010* had 12,906 nodes (330 diseases; 12,417 Ensembl genes; 122 Protein-Tissues; 37 RNA-Tissues) and 518,427 edges (34,201 hasDisease; 222,438 hasProteinExpression; 197,563 hasRNAExpression; 64,225 Interaction). Its edge density was 0.00311.

**FIGURE 2:**
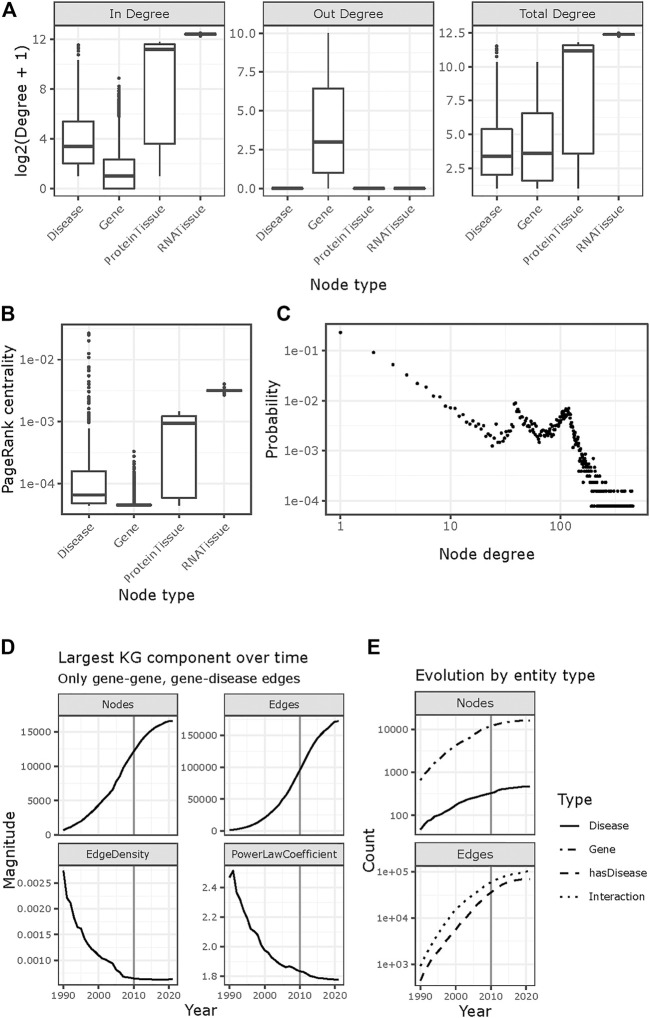
Topological properties of KG_Before 2010. **(A)** In- and out-degree of the nodes in each node type. Also shown is the total degree, defined as the sum of the in- and out-degree. All node types have hubs with over 100 edges (log2 (101) ≈ 6.7). **(B)** PageRank centrality, by node type. **(C)** Probability of each node degree suggest a power law; both axes are log scaled. **(D, E)** Temporal evolution of gene-gene and gene-disease links between 1990 and 2021. Edges were filtered according to their first mention in the literature. **(D)** Evolution of the largest weakly connected component over time, in terms of node count, edge count, edge density and power law coefficient. **(E)** Details on the relative growth by node types (genes or diseases) and by edge types (gene-gene interactions and gene-disease annotations).

The topological properties of *KG_Before2010* suggest it follows a scale free architecture (power law coefficient of 2, Figure 2C). Regarding their in-degree, genes are the least central nodes, followed by diseases, Protein-Tissues, and RNA-Tissues (Figure 2A). The out-degree is only positive for genes, with a maximum of 1,025. The total degree shows trends like those in the in-degree, except for genes and disease being on par due to the addition of the out-degree of genes. Using PageRank as a centrality measure depicts a similar scenario to the in-degree (Figure 2B). All the node types show heavy tails and hubs with more than 100 connections (Figures 2A,C). Such properties are in line with those of molecular networks and KGs in the biomedical domain.

### Temporal Evolution of Indications

To characterize the time dynamics of indication discovery, we started from the induced subgraph containing genes and diseases only and built year-specific subgraphs by removing the edges whose first mention in literature was posterior to the year under consideration (Figure 2). When accounting for all-time data (i.e., the 2021 network), the network encompassed 16,552 nodes and 172,118 edges (16,530 and 172,044 in the largest weakly connected component, respectively). In contrast, the largest connected component dating from 1990 consisted of 705 nodes and 1,360 edges, and the one from 2010 had 12,151 nodes and 95,375 edges. We observed a reduction of the increase rate in both the number of nodes and edges, more pronounced from 2015 onwards (Figure 2D), which might be explained by changes in the literature curation criteria or by the pace of data ingestion. Both edge density and the power law coefficient tend to decrease and plateau (Figure 2D), which might indicate the new addition of nodes over time that remain sparsely connected. The growth patterns in number of nodes and edges also hold for their sub-types (Figure 2E).

### Performance Evaluation of Hop-Based Methods

**FIGURE 3:**
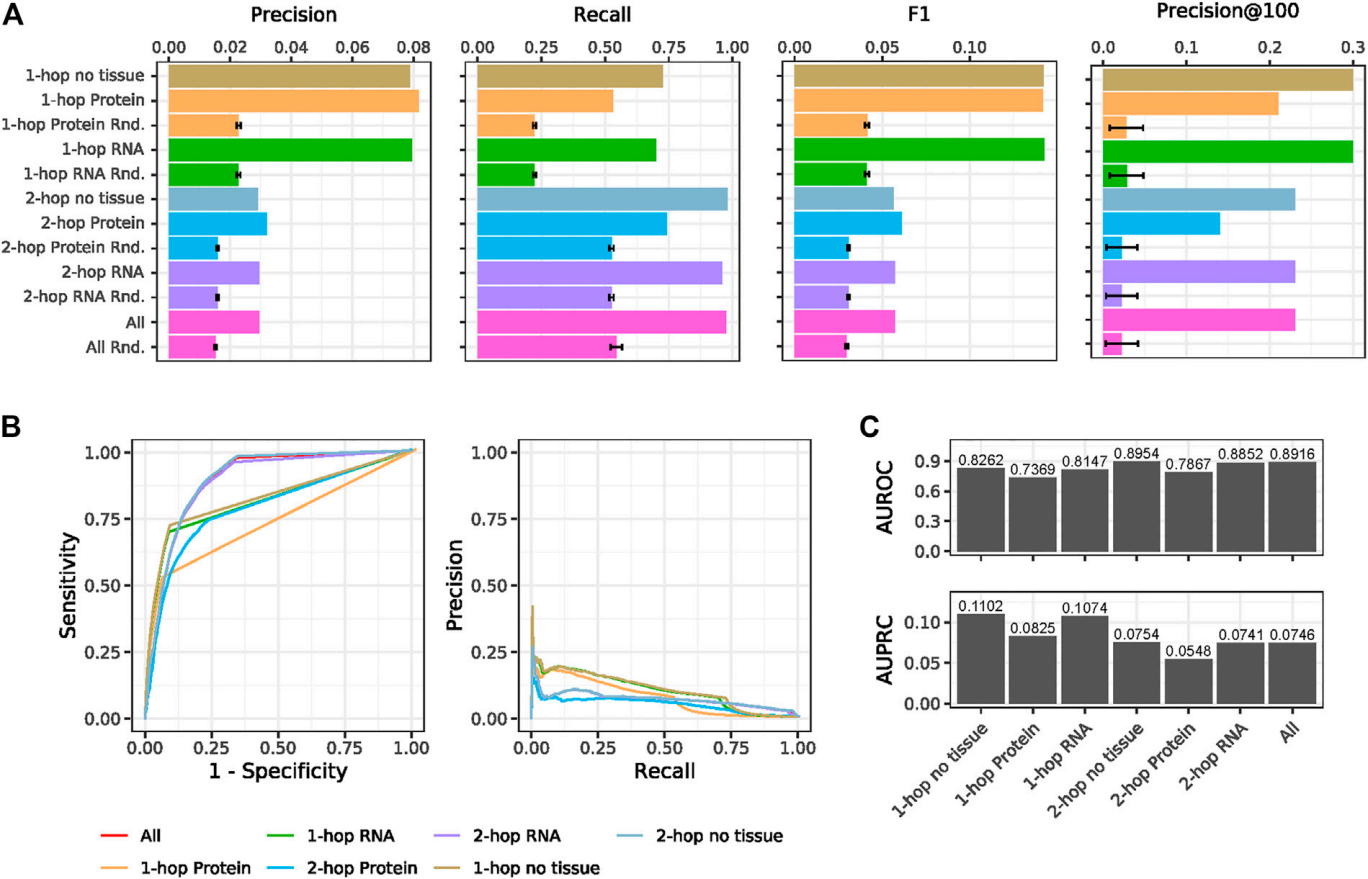
Performance evaluation of the hop-based predictions. **(A)** Precision, Recall, F1 and Precision@100 metrics calculated from all the gene-disease links predicted by each hop-based approach. **(B)** ROC and Precision-Recall performance curves for all the hop-based prediction methods. **(C)** Area under the ROC (AUROC) and Precision-Recall (AUPRC) curves shown in **(B)**.

Overall precision and recall values for the different strategies to predict indirect gene-disease links are shown in Table 3. The average performance metrics across 100 random KG are also reported, together with *p*-values from a one-sided z-test comparing the actual performance values and the distribution of random ones. In all cases, both precision and recall are higher than expected by chance with the one-hop with RNA tissue predictions producing the best precision-recall combination, followed by the one-hop with Protein tissue inferences (Table 3 and Figure 3A). Interestingly, while removing the tissue expression entity from the KG does have an impact on precision, the sensitivity of the one-hop and two-hop strategies without tissue is higher. This responds to the fact that, in these cases, the intermediary nodes connecting the gene with its predicted associated disease (see Figure 1B) do not have to be expressed in the same tissue, resulting in many more predicted gene-disease links and a higher probability to identify pairs in the gold standard. This is also the case for the two-hop and the union of all predictions (Figure 3A). However, when precision and recall are summarized with the F1 statistic, it becomes evident that the best predictions come from the one-hop methods (Figure 3A). We believe that, even though the F1 metric from the one-hop no tissue approach is comparable to that of the predictions with tissue constraints, it is better to ensure tissue homogeneity.

**TABLE 3:** Types of inferencing and their overall performance scores based on a total of 5,176 reference gene-disease links reported after 2010. Average ± standard deviations are reported for the random predictions.

Type of inferencing	Predicted links	Precision	Precision at100	Precision (random)	*p*-value precision	Recall	Recall (random)	*p*-value recall
All the inferences	170,506	0.0296	0.23	0.0152 ± 0.0003	2.55E-284	0.9737	0.5449 ± 0.0223	1.50E-81
One-hop and protein tissue	33,633	0.0817	0.21	0.0227 ± 0.0006	0.00E+00	0.5307	0.2234 ± 0.0060	0.00E+00
One-hop and RNA tissue	45,664	0.0794	0.3	0.0227 ± 0.0006	0.00E+00	0.7007	0.2235 ± 0.0061	0.00E+00
Two-hop and protein tissue	120,319	0.0319	0.14	0.0158 ± 0.0003	0.00E+00	0.7417	0.5247 ± 0.0088	4.50E-127
Two-hop and RNA tissue	167,939	0.0295	0.23	0.0157 ± 0.0003	7.10E-286	0.9571	0.5286 ± 0.0088	0.00E+00
One-hop without tissue	47,734	0.0787	0.30	0.0227 ± 0.0006	0.00E+00	0.7262	0.2235 ± 0.0061	0.00E+00
Two-hops without tissue	174,305	0.0291	0.23	0.0157 ± 0.0003	7.10E-286	0.9795	0.5286 ± 0.0088	0.00E+00

Table 3 shows that each hop-based method predicts tens of thousands of gene-disease links, a number of associations that is unlikely to be validated by experimental means. Therefore, we assessed the performance of the hop-based approaches for early retrieval by looking at metrics for the top-100 predictions (see Supplementary Table S1). In particular, Precision@100 shows that one-hop with RNA tissue and one-hop without tissue constraints are the best approaches for early recognition.

To better understand whether predicted gene-disease links with high scores were corroborated in publications after 2010, we built performance curves by scanning a list of all possible gene-disease pairs in KG_Before2010 separated by at most 2 hops (see Methods). Figure 3B shows the receiver operating characteristic (ROC) and Precision-Recall curves of all the gene-disease inference strategies, while Figure 3C shows the areas under these curves. The plots corroborate that one-hop predictions are the best when it comes to early retrieval and the tails of the curves represent the random ranks for gene-disease pairs that were given artificial scores of 0 (see Methods).

### Performance Evaluation Based on Random Forest on Several Knowledge Graph Embeddings

**FIGURE 4:**
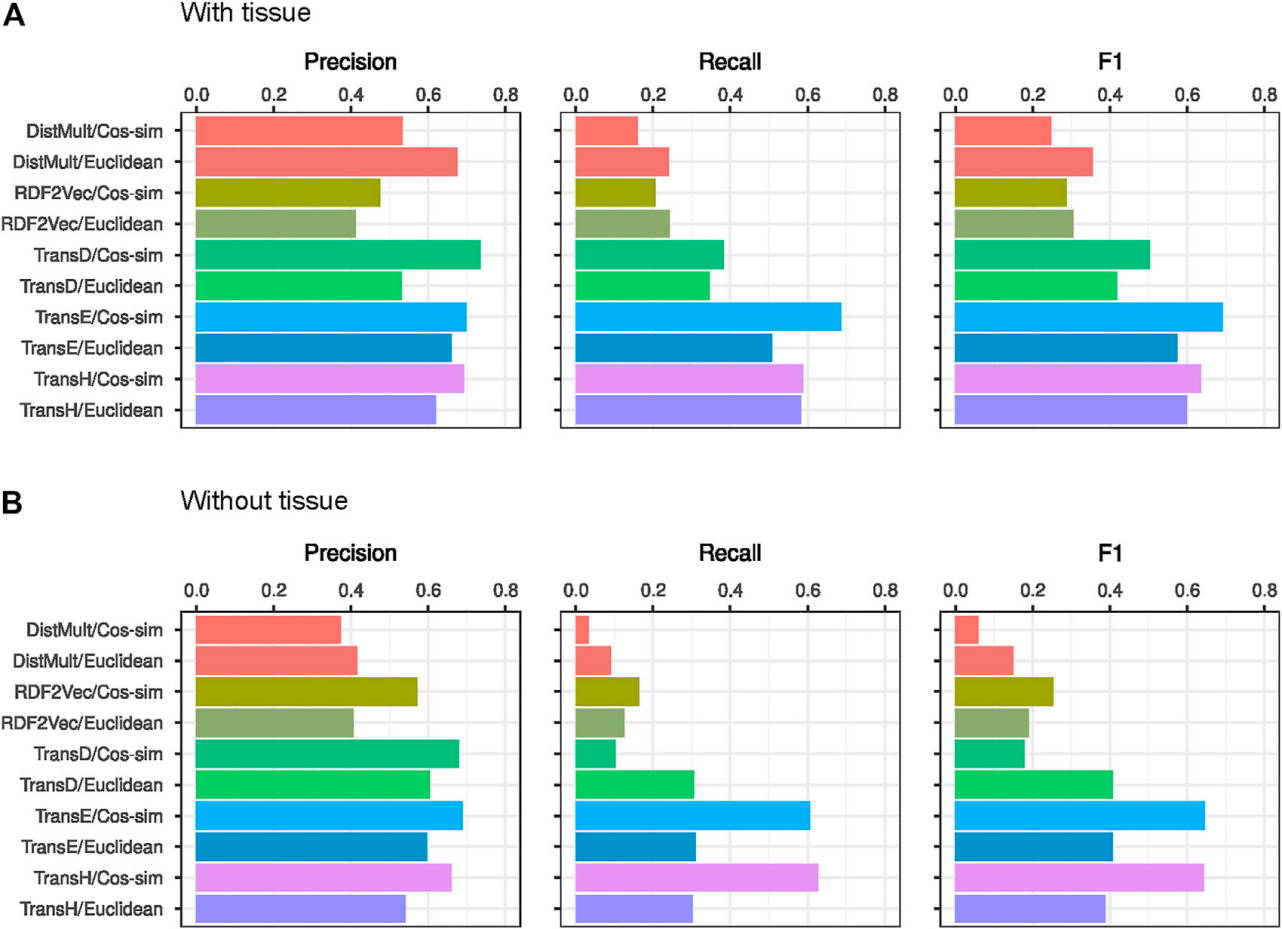
Embedding based gene-disease prediction evaluation. **(A)** Embedding performances in which gene-tissue links were included in the knowledge graph. **(B)** Embedding performances in which gene-tissue links were not included in the knowledge graph.

We employed 5 different dimensionality reduction strategies^15^ (Nunes et al., 2020) to embed the KG_Before2010 into a 200-dimensional space, obtain vector representations of genes and diseases, and use these vectors to build a machine learning model for gene-disease link prediction (see Methods and Figure 1C). Intuitively, a good embedding method should put gene-disease associations reported in the gold standard near each other in the latent space. We computed the distance between all gene-disease pairs, binned the distance range into 10 groups, and calculated the probability of finding gene-disease links reported after 2010 within each bin (Supplementary Figures S3, S4). This analysis showed that TransD, TransE, and TransH were the approaches that produced the expected gene-disease proximity patterns. To confirm whether these methods would indeed produce good gene-disease link predictions, we computed the Euclidean distances and cosine similarities between genes and diseases in the five different 200-dimensional spaces and used these measures to train a Random Forest model whose performance was evaluated with the gold standard mentioned above (see Methods). Table 4 and Figure 4 show the performance of the Random Forest predictions refined with and without the tissue expression information. TransE embeddings using cosine similarity vector as the training data for Random Forest achieved the best performance overall. These results also show that embeddings from the KG that contains gene-tissue links outperform the embeddings that don’t have this information, highlighting the importance of this entity for the embedding approaches (Table 4; Figure 4 and Supplementary Table S2).

**TABLE 4:** Random Forest predictions on different embeddings.

With tissue				No tissue			
Category	Precision	Recall	F1	Category	Precision	Recall	F1
DistMult/Cos-sim/@all	0.5339	0.1609	0.247278	DistMult_notissue/Cos-sim/@all	0.3747	0.0326	0.059981
DistMult/Euclidean/@all	0.6758	0.2413	0.355622	DistMult_notissue/Euclidean/@all	0.4152	0.0917	0.150222
RDF2Vec/Cos-sim/@all	0.4765	0.2057	0.287353	RDF2Vec_notissue/Cos-sim/@all	0.5711	0.1636	0.25434
RDF2Vec/Euclidean/@all	0.412	0.242	0.304905	RDF2Vec_notissue/Euclidean/@all	0.4074	0.1246	0.190835
TransD/Cos-sim/@all	0.7356	0.3827	0.503468	TransD_notissue/Euclidean/@all	0.6038	0.3066	0.40669
TransD/Euclidean/@all	0.5312	0.3462	0.419196	TransD_notissue/Cos-sim/@all	0.6794	0.1027	0.178428
TransE/Cos-sim/@all	**0.6988**	**0.6854**	**0.692035**	TransE_notissue/Cos-sim/@all	0.6894	0.6049	0.644392
TransE/Euclidean/@all	0.6604	0.5085	0.57458	TransE_notissue/Euclidean/@all	0.5958	0.3098	0.407639
TransH/Cos-sim/@all	0.6922	0.5884	0.636093	TransH_notissue/Euclidean/@all	0.54	0.3021	0.387446
TransH/Euclidean/@all	0.6187	0.5818	0.599683	TransH_notissue/Cos-sim/@all	0.6601	0.6263	0.642756

Bold numbers show the highest performance.

### Performance Evaluation Per Disease

Finally, we investigated the precision, recall, and F1 metrics for each disease separately to determine whether the biological knowledge encoded by the graph allows to make better predictions for certain diseases compared to others.

**FIGURE 5:**
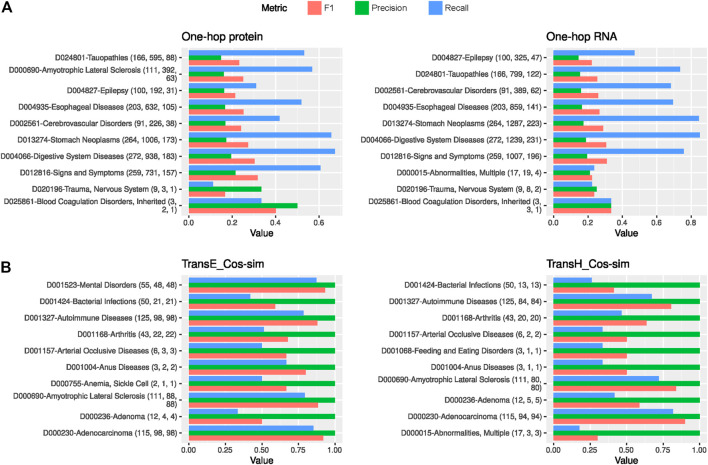
Performance evaluation per disease. **(A)** Precision, recall and F1 metrics attained by each the top two best performing hop-based prediction methods. **(B)** Same as **(A)** but for the top two embedding methods. Only the top 10 diseases are shown based on the precision value. The numbers in parentheses indicate the total number of gene-disease links in the gold standard for that disease, the number of predicted gene-disease links and how many of those were positive, respectively.

Figure 5A and Supplementary Figure S5 show that the hop-based strategies tend to perform well in a common set of disorders like Tauopathies (D024801), Esophageal Diseases (D004935), Stomach Neoplasms (D013274), and Digestive System Diseases (D004066). A similar pattern is observed for the embedding methods, with Arthritis (D01168), Amyotrophic Lateral Sclerosis (D000690), Mental Disorders (D001523), and Bacterial Infections (D001424) among the top-10 diseases in at least four embedding approaches (Figure 5B and Supplementary Figure S6).

The hop-based strategies show a similar behavior across diseases: many predictions, which makes recall high but causes low precision (see numbers in brackets in Supplementary Figure S5). Yet, the one-hop strategies show more balance between precision and recall than the two-hop strategies, as reflected by higher F1 scores (Supplementary Figure S5). In contrast, the embedding methods produced, in general, less predictions for the top-10 diseases, which led to low recalls but very high precisions as most of them were true hits (Supplementary Figure S6). This corroborates the metrics reported in Supplementary Table S2 and highlights that these prediction strategies are well suited for early retrieval tasks. Of note, when the embedding methods predicted more links for a disease (e.g., see Mental Disorders or ALS in TransE on Figure 5B), these were also mostly true hits, leading to high recall and F1 statistics. In the following section, we interpret some use cases for the diseases with the best local performance and showcase the interpretability of the predictions.

### Use Cases

In order to showcase the potential of our approach, we identified the best performing disease areas as promising domains of application. Then, we demonstrate how both predictions with highest literature support and with highest prediction score yield sensible links that were confirmed after 2010.

Based on Figure 4, the best performing embedding method was TransE followed by a Random Forest prediction on the cosine-similarity of the gene and disease low-dimensional vectors. Performance evaluation per disease (Figure 5B) showed that this method attained its highest F1 score for Mental Disorders (D001523) and Amyotrophic Lateral Sclerosis (ALS) (D000690). There were 55 gene-Mental Disorders pairs that were published after 2010, and with the TransE embedding strategy 48 of them were correctly predicted. In the ALS case, TransE recovered 88 of the 111 gene-disease pairs, TransH recovered 80, and the one-hop with Protein tissue strategy covered 63 reported after 2010. To explain these predictions, one can go back to the KG and study their paths, literature, and tissue support.

**FIGURE 6:**
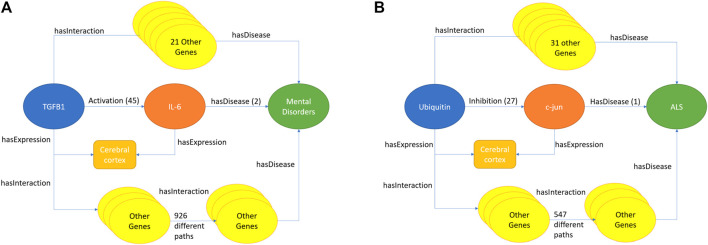
Example prediction from the knowledge graph. **(A)** TGFB1 is connected to Mental Disorders via IL6. **(B)** Ubiquitin is connected to ALS via c-Jun. Both panels also show the number of alternative connections from the genes to the predicted disease via one-hop and two-hop links.

For Mental Disorders, the path with the strongest literature backing (i.e., total number of publications) was the one linking TGFB1 with this disease group via IL-6, both genes co-expressed in the cerebral cortex (see Figure 6A). Before 2010, the activation of IL-6 by TGFB1 is endorsed by 45 publications, while the link between IL-6 and Mental Disorders is endorsed by 2 as shown in Figure 6A. Moreover, there are 22 different one-hop paths (TGFB1—gene X—Mental Disorders) and 926 different two-hops paths (TGFB1—gene Y—gene Z—Mental Disorders) between TGFB1 and Mental Disorders in which all genes are expressed in the cerebral cortex. The predicted TGFB1-Mental Disorders link, which later were published in López-González et al. (2019), supports the theory that dysfunction of the immune system plays an important role in the etiology of mental illnesses, such as schizophrenia and depression (Frydecka et al., 2013; Bialek et al., 2020). In fact, significantly higher serum levels of the IL-6 and TGFB1 cytokines have been reported in patients with schizophrenia compared to healthy controls (Ergün et al., 2017) and mutations in TGFB1 have been associated with the susceptibility and treatment response of schizophrenia (Frydecka et al., 2013) and major depressive disorder (Bialek et al., 2020).

For ALS, on the other hand, the top prediction from the embedding methods is Ubiquitin and ALS. There are 32 different one-hop paths (Ubiquitin—gene X—ALS) and 547 two-hops paths (Ubiquitin—gene Y—gene Z—ALS) in which all the genes in the paths were expressed in cerebral cortex as shown in Figure 6B. In this context, the strongest one-hop literature link (in terms of publication numbers) is Ubiquitin—c-Jun—ALS with 27 publications. The predicted Ubiquitin—ALS link is supported by the literature (Hasegawa and Arai, 2007; Watanabe et al., 2010; Keller et al., 2012) stating that Ubiquitin inclusions have been seen in ALS patients. JNK/c-Jun signaling has been found involved in the cell death caused by TDP-43, which is closely linked with ALS and ubiquitin inclusions (Suzuki and Matsuoka, 2013). It is important to note that the link between Ubiquitin and ALS has been discussed in the literature before 2010 (Hasegawa and Arai, 2007), but this was not considered a high-confidence association in Metabase and was therefore not known by our predictive model.

## Discussion

In this study, we presented the evaluation of the effectiveness of the methodology that we developed to build a comprehensive KG for target-repurposing (indication expansion) studies. We first evaluated the effectiveness of the constructed KG for target-disease prediction via semantic inferencing, i.e., by linking targets and diseases that are one or two hops away from each other passing through genes that are expressed in the same tissue as the target. In addition, we checked whether embedding our KG to a low dimensional space to then use the inferred gene and disease coordinates to generate dis-/similarity inputs for a machine learning model could lead to more reliable predictions. For these experiments, we divided the KG in two parts such that edges reported before 2010 were used as training data and edges reported after 2010 served as our gold standard. This splitting allowed us to have a reliable gold standard reference, supported by the literature.

Our experiments showed that the hop-based strategies using RNA- and Protein-level expression data significantly outperformed our random baselines and were more precise than hop-based predictors without tissue information. Also, the one-hop RNA prediction method outperformed the two-hop and the one-hop Protein strategies. This reflects the fact that there is much more available information about gene expression at the RNA level (and/or protein abundance data is still incomplete) and suggests that two-hop predictions incorporate too many false positives to be reliable, especially for early recognition. In addition, using Euclidean distances and cosine similarities between gene and disease vectors inferred by KG embeddings to train a Random Forest model led to much better gene-disease prediction results. In particular, the TransE and TransH embedding methods followed by the computation of cosine similarities between genes and diseases represented the best training platform for the constructed Random Forests. Our initial quality controls of the embeddings already hinted at this result, as the probability of finding gold standard gene-disease associations at short embedding distances was very high for these methods. Moreover, added value by gene-tissue links is more visible in the KG Embeddings strategies.

One of the limitations of this study is that when creating the training data set, the true negatives are usually unknown. We use as proxy gene-diseases for which no connection is known, but this does not imply that they are unrelated. This can also overestimate the number of false positives: even though a predicted link might have not yet discovered, we simply assumed that if the predicted link does not appear in the KG after 2010, then it is a false positive. Secondly, gene-gene interaction network is incomplete due to evolution of the network over time (which is continuous), and also it is technically challenging and costly to test each protein pairs’ interaction in humans. Thirdly, we have the relations for tissue-specific expressions, but we cannot distinguish cell type-specific effects. And genes and diseases which are linked to low number of genes and diseases (in other words with less neighbors) are most likely result in worse predictions. Lastly, this study only focuses on the human data and other organisms are out of scope. However, this method can be applied on other organism data as well.

Although the explainability of the predictions, i.e., the glass-box property of the KG, is easier to see in the hop-based methods, it is also possible to query the KG in order to explain the predictions produced by embedding combined with machine learning approaches, as we did for our two use cases. In addition, it is possible to inspect the resulting Random Forest model to determine which features have a strong impact on a decision. This kind of analysis was outside of the scope of this study.

To the best of our knowledge, this work is the first one to apply inferencing constrained by tissue expression on a semantic KG. Moreover, our KG is built from full-text literature sources and not only abstracts, which means that the graph does not miss any important information and does not depend on NLP tools like other literature-based approaches. As future work, we plan to extend the data sources employed to construct our KG, explore other predictive modelling methods, as well as to make it a key component of our target identification pipelines.

## Data Availability

The data analyzed in this study was obtained from Cortellis Metabase, the following licenses/restrictions apply: it is a commercial database. Code used throughout this study is available at https://github.com/bi-compbio/kg_for_ie. Any further requests to access these datasets should be directed to OG, ozge.gurbuz@boehringer-ingelheim.com

## Footnotes

1
https://www.disgenet.org/(accessed on 12.11.21).

2
https://rdflib.readthedocs.io/en/stable/apidocs/rdflib.html (accessed on 12.11.21).

3
https://rdf4j.org/documentation/programming/rdfstar/(accessed on 12.11.21).

4
https://www.cortellislabs.com/page/?api=api-MB (accessed on 12.11.21).

5
https://www.proteinatlas.org/(accessed on 12.11.21).

6
https://github.com/IBCNServices/pyRDF2Vec (accessed on 11.10.21).

7
https://github.com/thunlp/OpenKE (accessed on 11.10.21).

8
https://github.com/thunlp/OpenKE (accessed on 11.10.21).

9
https://github.com/liseda-lab/KGE_Predictions_GD (accessed on 11.10.21).

10
https://github.com/liseda-lab/KGE_Predictions_GD (accessed on 11.10.21).

11
https://www.cortellislabs.com/page/?api=api-MB (accessed on: 09.11.2021).

12
https://string-db.org/(accessed on: 12.11.2021).

13
https://www.disgenet.org/(accessed on: 12.11.2021).

14
https://bioconductor.org/packages/release/bioc/vignettes/hpar/inst/doc/hpar.html (Accessed on: 13.10.2021).

15
https://github.com/liseda-lab/KGE_Predictions_GD (Accessed on 13.10.2021).
