# Supplementary material for: Knowledge Graphs for Indication Expansion: An Explainable Target-Disease Prediction Method
Source: Front Genet. 2022 Mar 14;13:814093. doi: 10.3389/fgene.2022.814093 (PMC8963915; doi:10.3389/fgene.2022.814093)
Supplement: Supplementary file 1 [file DataSheet1.docx]

Supplementary Material


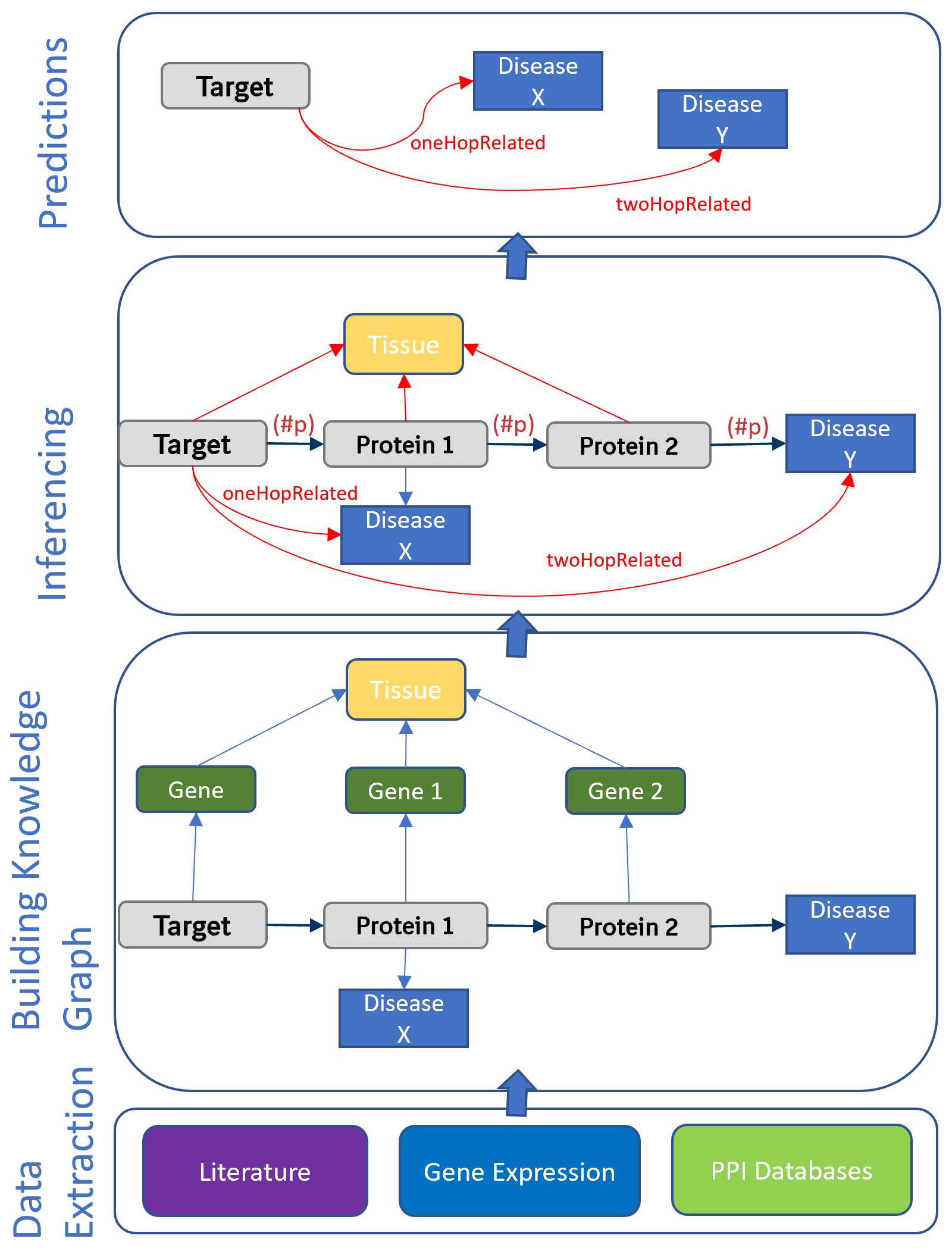


Supplementary Figure 1 Pipeline for knowledge graph building and analysis. From bottom to top: possible data extraction options, way in which entities can be linked to build the Knowledge Graph, inferencing of new target-disease links based on one- or two-hop separation between the target and disease via one or two proteins that are expressed in the same tissue as the target (the number of PubMed IDs #p associated with each link in the graph can be used to rank the predictions), possible indirect link predictions.


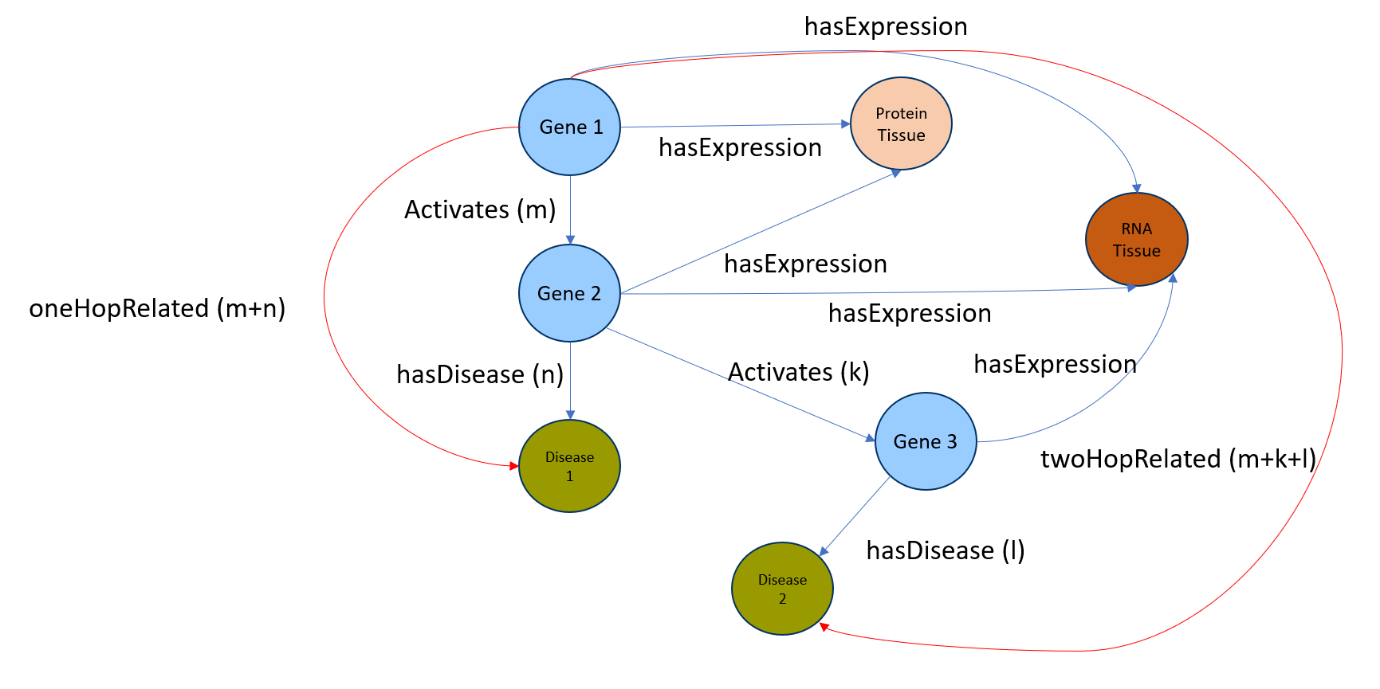


Supplementary Figure 2 Example knowledge graph. Letters in parenthesis represent the number of publications associated with each link. Gene 1 is one-hop related to Disease 1 via Gene2 because they are both linked to the same protein tissue expression node. And this one hop relation is weighted as the sum of the number of publications reporting the link between gene1-gene2 and gene2-disease1. Additionally, Gene 1 is two-hop related to Disease 2 via Gene 2 and Gene 3 because they share the same RNA tissue expression node.

Supplementary Table 1 Performance evaluation for the top-100 predictions

| **Type of inferencing** | **Precision @100** | **Recall @100 Hit** | **F1 @100 Hit** |
| --- | --- | --- | --- |
| All the inferences | 0.23 | 0.0044 | 0.0087 |
| One-hop and protein tissue | 0.21 | 0.0041 | 0.0080 |
| One-hop and RNA tissue | 0.3 | 0.0058 | 0.0114 |
| Two-hop and protein tissue | 0.14 | 0.0027 | 0.0053 |


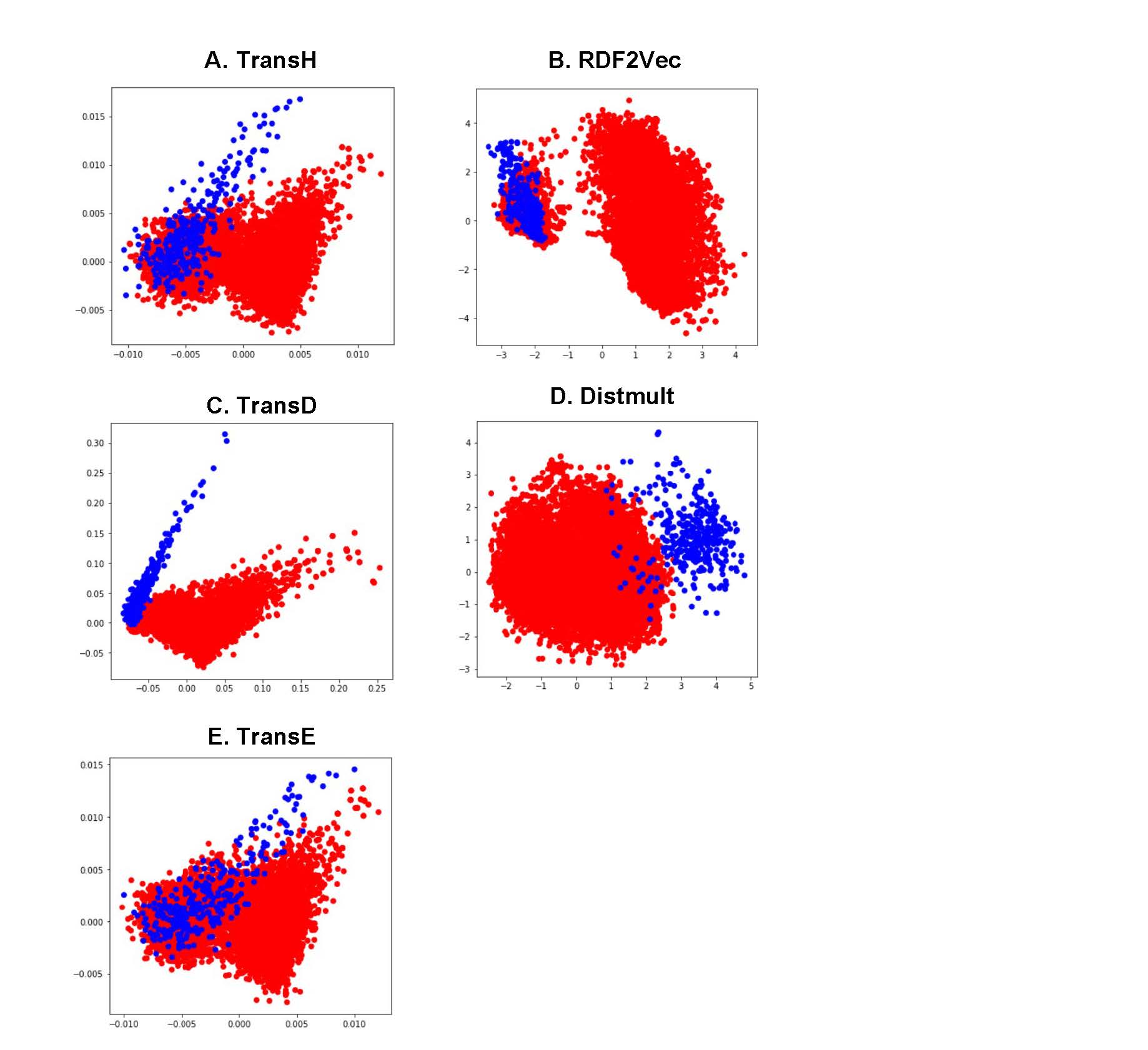


Supplementary Figure 3. Two-dimensional representation of the embedding results. Red dots are genes, while blue dots are diseases.


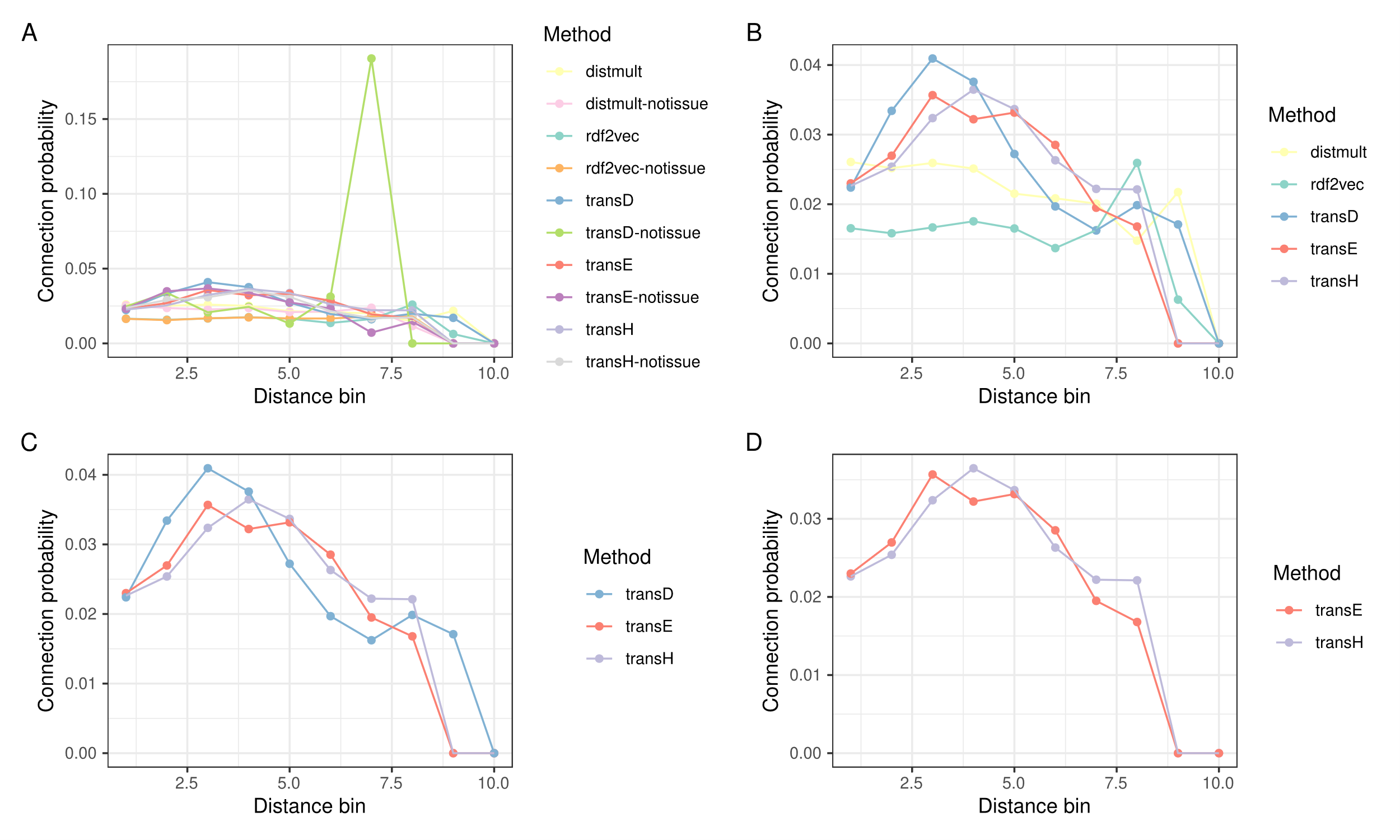


Supplementary Figure 4 Connection probability plots for the different knowledge graph embedding methods. These plots show the probability of finding a gene-disease pair that is connected in the gold standard knowledge graph based on low-dimensional coordinates inferred with KG_Before2010. The probabilities are shown for 10 different distance bins. (A) All embedding strategies with and without tissue information. (B) Embedding strategies with tissue information. (C) TransD, TransE, and TransH embeddings. (D) TransE and TransH embeddings.

Supplementary Table 2 Performance results for Random Forest applied to the results of the different embedding methods

| category | precision | recall | f1 | category | precision | recall | f1 |
| --- | --- | --- | --- | --- | --- | --- | --- |
| DistMult/Cos-sim/@100 | 0.17 | 0.0033 | 0.0064 | DistMult_notissue/Cos-sim/@100 | 0.03 | 6.00E-04 | 0.0011 |
| DistMult/Cos-sim/@25 | 0.2 | 0.001 | 0.0019 | DistMult_notissue/Cos-sim/@25 | 0.04 | 2.00E-04 | 4.00E-04 |
| DistMult/Cos-sim/@50 | 0.2 | 0.0019 | 0.0038 | DistMult_notissue/Cos-sim/@50 | 0.04 | 4.00E-04 | 8.00E-04 |
| DistMult/Cos-sim/@75 | 0.16 | 0.0023 | 0.0046 | DistMult_notissue/Cos-sim/@75 | 0.0267 | 4.00E-04 | 8.00E-04 |
| DistMult/Cos-sim/@all | 0.5339 | 0.1609 | 0.247278 | DistMult_notissue/Cos-sim/@all | 0.3747 | 3.26E-02 | 0.059981 |
| DistMult/Euclidean/@100 | 0.25 | 0.0048 | 0.0095 | DistMult_notissue/Euclidean/@100 | 0.07 | 0.0014 | 0.0027 |
| DistMult/Euclidean/@25 | 0.32 | 0.0015 | 0.0031 | DistMult_notissue/Euclidean/@25 | 0.08 | 4.00E-04 | 8.00E-04 |
| DistMult/Euclidean/@50 | 0.3 | 0.0029 | 0.0057 | DistMult_notissue/Euclidean/@50 | 0.08 | 8.00E-04 | 0.0015 |
| DistMult/Euclidean/@75 | 0.2533 | 0.0037 | 0.0072 | DistMult_notissue/Euclidean/@75 | 0.0667 | 0.001 | 0.0019 |
| DistMult/Euclidean/@all | **0.6758** | **0.2413** | **0.355622** | DistMult_notissue/Euclidean/@all | 0.4152 | 0.0917 | 0.150222 |
| RDF2Vec/Cos-sim/@100 | 0.19 | 0.0037 | 0.0072 | RDF2Vec_notissue/Cos-sim/@100 | 0.14 | 0.0027 | 0.0053 |
| RDF2Vec/Cos-sim/@25 | 0.16 | 8.00E-04 | 0.0015 | RDF2Vec_notissue/Cos-sim/@25 | 0.08 | 4.00E-04 | 8.00E-04 |
| RDF2Vec/Cos-sim/@50 | 0.18 | 0.0017 | 0.0034 | RDF2Vec_notissue/Cos-sim/@50 | 0.18 | 0.0017 | 0.0034 |
| RDF2Vec/Cos-sim/@75 | 0.2 | 0.0029 | 0.0057 | RDF2Vec_notissue/Cos-sim/@75 | 0.1467 | 0.0021 | 0.0042 |
| RDF2Vec/Cos-sim/@all | **0.4765** | **0.2057** | **0.287353** | RDF2Vec_notissue/Cos-sim/@all | 0.5711 | 0.1636 | 0.25434 |
| RDF2Vec/Euclidean/@100 | 0.24 | 0.0046 | 0.0091 | RDF2Vec_notissue/Euclidean/@100 | 0.1 | 0.0019 | 0.0038 |
| RDF2Vec/Euclidean/@25 | 0.2 | 0.001 | 0.0019 | RDF2Vec_notissue/Euclidean/@25 | 0.04 | 2.00E-04 | 4.00E-04 |
| RDF2Vec/Euclidean/@50 | 0.18 | 0.0017 | 0.0034 | RDF2Vec_notissue/Euclidean/@50 | 0.14 | 0.0014 | 0.0027 |
| RDF2Vec/Euclidean/@75 | 0.2267 | 0.0033 | 0.0065 | RDF2Vec_notissue/Euclidean/@75 | 0.1067 | 0.0015 | 0.003 |
| RDF2Vec/Euclidean/@all | 0.412 | 0.242 | 0.304905 | RDF2Vec_notissue/Euclidean/@all | 0.4074 | 0.1246 | 0.190835 |
| TransD/Cos-sim/@100 | 0.45 | 0.0087 | 0.0171 | TransD_notissue/Euclidean/@25 | 0.4 | 0.0019 | 0.0038 |
| TransD/Cos-sim/@25 | 0.64 | 0.0031 | 0.0062 | TransD_notissue/Euclidean/@50 | 0.44 | 0.0043 | 0.0084 |
| TransD/Cos-sim/@50 | 0.54 | 0.0052 | 0.0103 | TransD_notissue/Euclidean/@75 | 0.4133 | 0.006 | 0.0118 |
| TransD/Cos-sim/@75 | 0.48 | 0.007 | 0.0137 | TransD_notissue/Euclidean/@100 | 0.41 | 0.0079 | 0.0155 |
| TransD/Cos-sim/@all | **0.7356** | **0.3827** | **0.503468** | TransD_notissue/Euclidean/@all | 0.6038 | 0.3066 | 0.40669 |
| TransD/Euclidean/@100 | 0.36 | 0.007 | 0.0136 | TransD_notissue/Cos-sim/@25 | 0 | 0 | NA |
| TransD/Euclidean/@25 | 0.28 | 0.0014 | 0.0027 | TransD_notissue/Cos-sim/@50 | 0.06 | 6.00E-04 | 0.0011 |
| TransD/Euclidean/@50 | 0.32 | 0.0031 | 0.0061 | TransD_notissue/Cos-sim/@75 | 0.0933 | 0.0014 | 0.0027 |
| TransD/Euclidean/@75 | 0.32 | 0.0046 | 0.0091 | TransD_notissue/Cos-sim/@100 | 0.1 | 0.0019 | 0.0038 |
| TransD/Euclidean/@all | 0.5312 | 0.3462 | 0.419196 | TransD_notissue/Cos-sim/@all | 0.6794 | 0.1027 | 0.178428 |
| TransE/Cos-sim/@100 | 0.74 | 0.0143 | 0.0281 | TransE_notissue/Cos-sim/@100 | 0.61 | 0.0118 | 0.0231 |
| TransE/Cos-sim/@25 | 0.68 | 0.0033 | 0.0065 | TransE_notissue/Cos-sim/@25 | 0.72 | 0.0035 | 0.0069 |
| TransE/Cos-sim/@50 | 0.72 | 0.007 | 0.0138 | TransE_notissue/Cos-sim/@50 | 0.7 | 0.0068 | 0.0134 |
| TransE/Cos-sim/@75 | 0.7067 | 0.0102 | 0.0202 | TransE_notissue/Cos-sim/@75 | 0.6533 | 0.0095 | 0.0187 |
| TransE/Cos-sim/@all | **0.6988** | **0.6854** | **0.692035** | TransE_notissue/Cos-sim/@all | 0.6894 | 0.6049 | 0.644392 |
| TransE/Euclidean/@100 | 0.48 | 0.0093 | 0.0182 | TransE_notissue/Euclidean/@100 | 0.33 | 0.0064 | 0.0125 |
| TransE/Euclidean/@25 | 0.44 | 0.0021 | 0.0042 | TransE_notissue/Euclidean/@25 | 0.2 | 0.001 | 0.0019 |
| TransE/Euclidean/@50 | 0.5 | 0.0048 | 0.0096 | TransE_notissue/Euclidean/@50 | 0.28 | 0.0027 | 0.0054 |
| TransE/Euclidean/@75 | 0.48 | 0.007 | 0.0137 | TransE_notissue/Euclidean/@75 | 0.3067 | 0.0044 | 0.0088 |
| TransE/Euclidean/@all | 0.6604 | 0.5085 | 0.57458 | TransE_notissue/Euclidean/@all | 0.5958 | 0.3098 | 0.407639 |
| TransH/Cos-sim/@100 | 0.62 | 0.012 | 0.0235 | TransH_notissue/Euclidean/@25 | 0.32 | 0.0015 | 0.0031 |
| TransH/Cos-sim/@25 | 0.8 | 0.0039 | 0.0077 | TransH_notissue/Euclidean/@50 | 0.32 | 0.0031 | 0.0061 |
| TransH/Cos-sim/@50 | 0.62 | 0.006 | 0.0119 | TransH_notissue/Euclidean/@75 | 0.2933 | 0.0043 | 0.0084 |
| TransH/Cos-sim/@75 | 0.6133 | 0.0089 | 0.0175 | TransH_notissue/Euclidean/@100 | 0.3 | 0.0058 | 0.0114 |
| TransH/Cos-sim/@all | **0.6922** | **0.5884** | **0.636093** | TransH_notissue/Euclidean/@all | 0.54 | 0.3021 | 0.387446 |
| TransH/Euclidean/@100 | 0.41 | 0.0079 | 0.0155 | TransH_notissue/Cos-sim/@25 | 0.72 | 0.0035 | 0.0069 |
| TransH/Euclidean/@25 | 0.6 | 0.0029 | 0.0058 | TransH_notissue/Cos-sim/@50 | 0.66 | 0.0064 | 0.0126 |
| TransH/Euclidean/@50 | 0.52 | 0.005 | 0.01 | TransH_notissue/Cos-sim/@75 | 0.64 | 0.0093 | 0.0183 |
| TransH/Euclidean/@75 | 0.4267 | 0.0062 | 0.0122 | TransH_notissue/Cos-sim/@100 | 0.65 | 0.0126 | 0.0246 |
| TransH/Euclidean/@all | 0.6187 | 0.5818 | 0.599683 | TransH_notissue/Cos-sim/@all | 0.6601 | 0.6263 | 0.642756 |


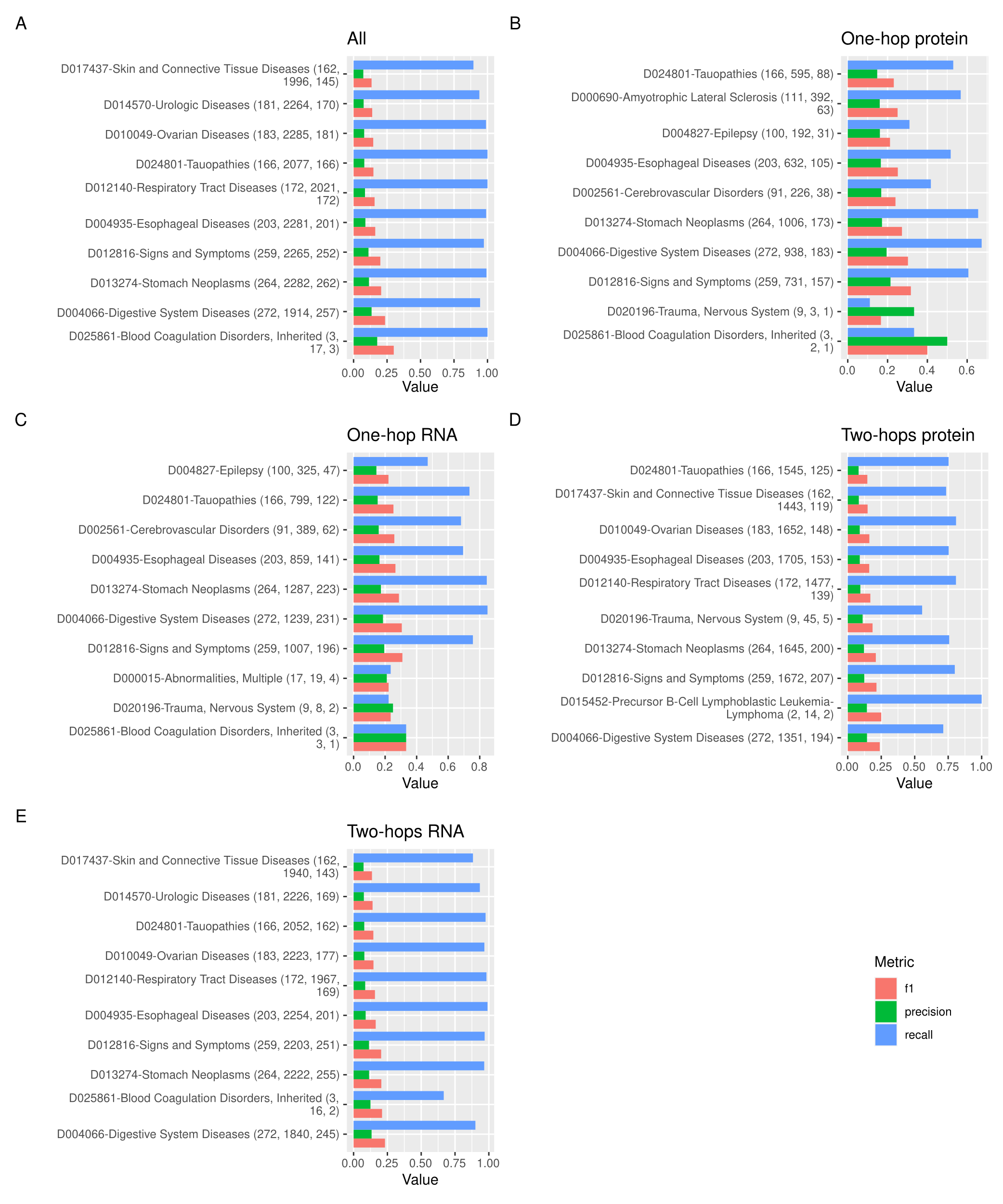


Supplementary Figure 5 Performance evaluation per disease for the hop-based prediction methods. (A-E) Precision, recall and F1 attained by each one of the different hop-based inference strategies per disease. Only the top 10 diseases are shown based on the precision value. The numbers in parenthesis indicate the total number of gene-disease links in the gold standard for that disease, the number of predicted gene-disease links and how many of those were positive, respectively.


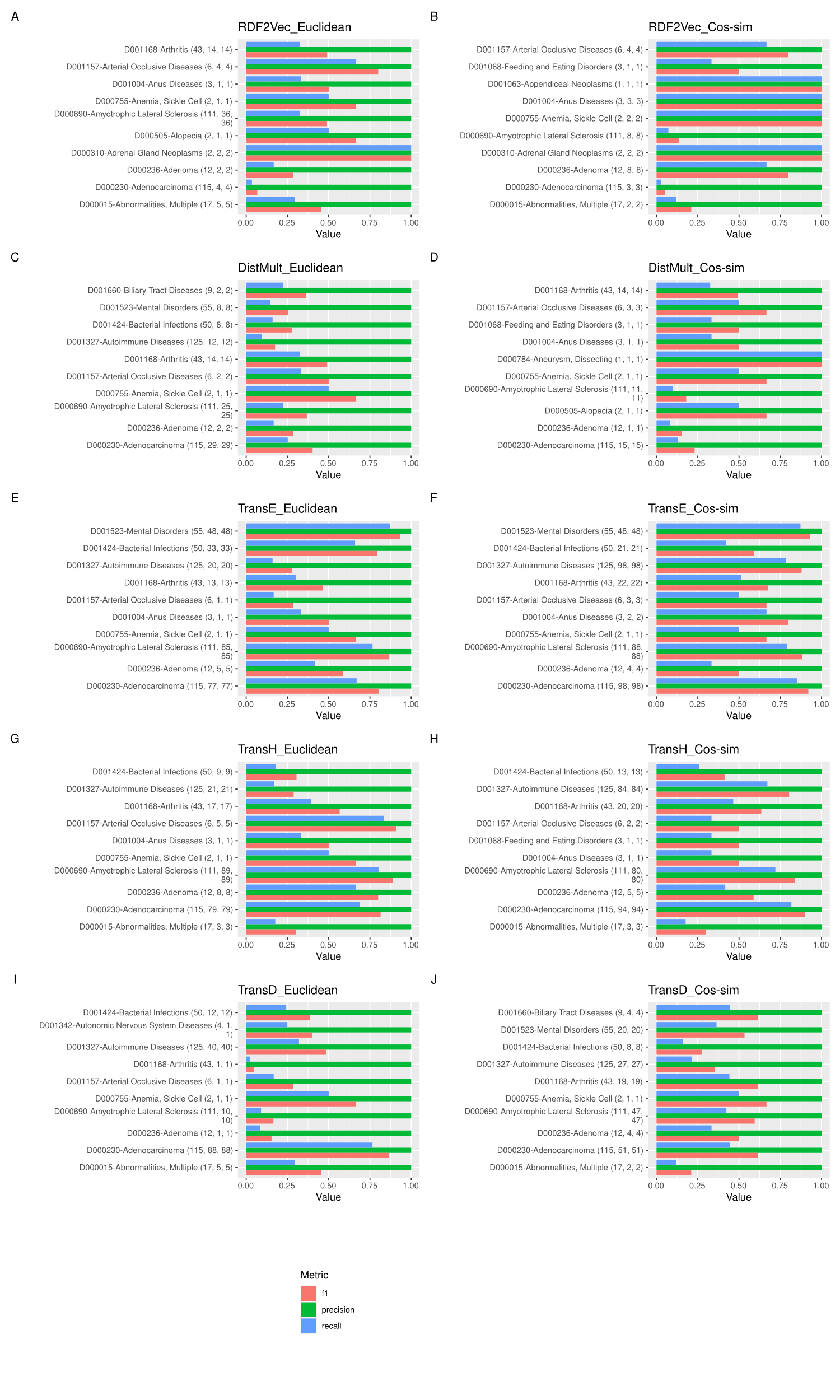


Supplementary Figure 6 Performance evaluation per disease for predictions based on Random Forest applied to different embeddings outcomes. (A-J) Precision, recall and F1 attained by each one of the different embedding strategies per disease. Only the top 10 diseases are shown based on the precision value. The numbers in parenthesis indicate the total number of gene-disease links in the gold standard for that disease, the number of predicted gene-disease links and how many of those were positive, respectively.
